# Cumulative live birth rates under three consecutive IVF/ICSI treatment cycles are reduced in women with endometriosis and/or adenomyosis diagnosed by ultrasonography

**DOI:** 10.1093/humrep/deaf184

**Published:** 2025-09-20

**Authors:** Sara Alson, Amelie Stenqvist, Povilas Sladkevicius

**Affiliations:** Obstetric, Gynecological and Prenatal Ultrasound Research, Department of Clinical Sciences, Lund University, Malmö, Sweden; Department of Obstetrics and Gynecology, Skåne University Hospital, Malmö, Sweden; Reproductive Medicine Center, Skåne University Hospital, Malmö, Sweden; Reproductive Medicine Center, Skåne University Hospital, Malmö, Sweden; Obstetric, Gynecological and Prenatal Ultrasound Research, Department of Clinical Sciences, Lund University, Malmö, Sweden; Department of Obstetrics and Gynecology, Skåne University Hospital, Malmö, Sweden

**Keywords:** infertility, subfertility, assisted reproductive treatment, ART, endometriosis, adenomyosis, ultrasound, MUSA, IDEA

## Abstract

**STUDY QUESTION:**

Does endometriosis and/or adenomyosis, diagnosed using the International Deep Endometriosis Analysis (IDEA) group and the Morphological Uterus Sonographic Assessment (MUSA) group revised definitions, impact cumulative live birth rates (CLBR) after three consecutive IVF or ICSI treatments?

**SUMMARY ANSWER:**

Women with endometriosis and/or adenomyosis, as diagnosed using transvaginal ultrasonography, had a 15% reduced chance of having a cumulative live birth after three consecutive IVF/ICSI treatments compared to women without these conditions.

**WHAT IS KNOWN ALREADY:**

Women with endometriosis or adenomyosis reportedly have lower live birth rates after their first IVF/ICSI treatment. However, most women undergo multiple cycles, and given their shared pathophysiology, the combined impact of both conditions over consecutive treatments remains unclear.

**STUDY DESIGN, SIZE, DURATION:**

This was a prospective cohort study of 1035 women undergoing up to three consecutive IVF/ICSI treatments at a university hospital between January 2019 and April 2024. Swedish regulations entitle women to up to three subsidized treatment cycles, including fresh and/or frozen embryo transfers, until the birth of a living child is achieved.

**PARTICIPANTS/MATERIALS, SETTING, METHODS:**

All 1035 included women underwent a transvaginal ultrasound examination prior to starting their first treatment. Using the IDEA and revised MUSA definitions, respectively, in total 293 (28.3%) women had endometriosis and/or direct features of adenomyosis on ultrasonography. All 1035 women underwent the first treatment cycle. In total, 818 (79.0%) women [595 (80.2%) of women without endometriosis and/or adenomyosis and 223 (76.1%) of women with either of the diseases] underwent all treatments they were eligible for. A total of 217 (21.0%) women dropped out after the first or second treatment even if they had not achieved a live birth. In total, 1725 fresh treatment cycles were initiated, leading to 1283 fresh and 622 frozen embryo transfers. Live births were recorded. The adjusted relative risk (aRR) for cumulative live birth after three consecutive IVF/ICSI treatment cycles was calculated on an intention-to-treat (ITT) as well as per-protocol (PP) basis, using a modified Poisson regression analysis, adjusting for age as a potential confounder.

**MAIN RESULTS AND THE ROLE OF CHANCE:**

The CLBR over three consecutive IVF/ICSI treatment cycles was 666/818 (81.4%) in the total cohort. In an ITT and PP analyses, respectively, women with endometriosis and/or adenomyosis had a lower CLBR of 156/293 (53.2%) or 156/223 (70.0%) compared to women without, CLBR of 510/742 (68.7%) or 510/595 (85.7%), *P* < 0.001. The aRR for cumulative live birth for women with endometriosis and/or adenomyosis was aRR (ITT) 0.80 (95% CI, 0.71–0.90), *P* < 0.001, and aRR (PP) 0.85 (95% CI, 0.77–0.93), *P* < 0.001 compared to women without the diseases. After stratifying the results per treatment cycle, the LBR after the first treatment for women with endometriosis and/or adenomyosis was 90/293 (30.7%), aRR 0.69 (95% CI 0.57–0.84), *P* < 0.001, after the second 44/154 (28.6%), aRR 0.72 (95% CI 0.54–0.96), *P* = 0.023, and after the third treatment 22/84 (26.2%), aRR 0.83 (95% CI 0.54–1.28), *P* = 0.183. For women without the diseases, the LBR was 335/742 (45.1%) in the first cycle, 132/319 (41.4%) in the second, and 43/133 (32.3%) in the third cycle. The largest differences were seen after fresh compared to frozen embryo transfers.

**LIMITATIONS, REASONS FOR CAUTION:**

The ultrasound examinations were performed at a tertiary care hospital by an examiner with expertise in endometriosis and adenomyosis. According to the revised MUSA definitions, direct features of adenomyosis are pathognomonic, whereas indirect features are only indicative of the disease. It is possible that some women with only indirect features, who were considered healthy in this study, in fact had the disease and therefore were wrongly classified.

**WIDER IMPLICATIONS OF THE FINDINGS:**

Despite a lower CLBR over three IVF/ICSI cycles, women with endometriosis and/or adenomyosis still have a reasonable chance of achieving a live birth with consecutive treatments. Negative results after the first treatment should not be an argument to withhold further attempts. Future research should explore strategies to enhance treatment success in this population, including the role of long-term suppression protocols, exogenous progesterone dosing, and personalized embryo transfer approaches.

**STUDY FUNDING/COMPETING INTEREST(S):**

This study was supported by regional research grants from Region Skåne, Sweden.

**TRIAL REGISTRATION NUMBER:**

N/A.

## Introduction

Endometriosis and adenomyosis are estrogen-dependent, closely related and often co-existing conditions, characterized by ectopic endometrial glands and stroma outside the uterine cavity (Bulun, 2009; Bulun *et al.*, 2023). Ectopic endometrial tissue is associated with inflammation, local hyperestrogenism, progesterone-resistance, and aberrant angiogenesis—factors that may contribute to subfertility (Bulun *et al.*, 2010; Broi *et al.*, 2019). Endometriosis and adenomyosis are estimated to affect 10–20% of reproductive-age women and 20–50% of those undergoing ART (Giudice and Kao, 2004). Previous studies, including our own, have reported lower live birth rates (LBR) after the first IVF or ICSI cycle in women with either endometriosis or adenomyosis (Alson *et al*., 2024a,b). While both conditions may independently impair ART success, their combined effect remains less well understood (Vercellini *et al.*, 2023). Given their shared pathophysiology and inflammation-driven effects on reproduction, some researchers propose that endometriosis and adenomyosis may represent different phenotypes of the same disease (Bulun *et al.*, 2023; Donnez *et al.*, 2024). Therefore, when evaluating their effect on ART outcomes, it may be necessary to look at both diseases simultaneously (Cozzolino *et al.*, 2025).

Most studies assess IVF/ICSI outcomes using per-cycle pregnancy or LBR, rather than cumulative live birth rates (CLBR) over multiple treatment attempts. However, many women who do not conceive after their first IVF/ICSI cycle pursue additional treatment attempts. Clinical management often involves protocol adjustments across subsequent cycles, to maximize the chances of success. For those undergoing multiple cycles, these adjustments may help counteract the negative effects of endometriosis and adenomyosis observed in the first cycle. Therefore, CLBR over several cycles would provide a more accurate estimate of the impact of endometriosis and adenomyosis on ART outcomes than rates based on a first, single treatment cycle.

Clearly defined ultrasonographic criteria are prerequisites for establishing a correct diagnosis and for studying the potential impact of the diseases on ART outcomes. To harmonize the terminology used to describe endometriosis and adenomyosis on transvaginal ultrasonography (TVUS), and to facilitate comparison between studies, the International Deep Endometriosis Analysis (IDEA) group (Guerriero *et al.*, 2016) and the Morphological Uterus Sonographic Assessment (MUSA) group (Van den Bosch *et al.*, 2015, 2019; Harmsen *et al.*, 2022) have suggested standardized ultrasound criteria.

This study aims to evaluate the CLBR over three consecutive IVF/ICSI cycles, including fresh (ET) and frozen embryo transfers (FET), in women with and without endometrioma, deep endometriosis (DE), and/or adenomyosis (E/A) according to the IDEA (Guerriero *et al.*, 2016) and revised MUSA definitions (Harmsen *et al.*, 2022), respectively.

## Materials and methods

### Study population and design

This was an observational prospective cohort study at the Reproductive Medicine Centre (RMC) at Skane University Hospital, Malmö, Sweden. Women scheduled for their first ART were included in the study between December 2018 and May 2021. In Sweden, women are eligible for publicly subsidized ART if they meet the following criteria: non-smoking women aged 25 to ≤39 years with a normal BMI (18 to ≤30 kg/m^2^), with ≥1 year’s infertility and no common children with the present partner. Women are offered up to three IVF/ICSI treatment cycles, until the birth of a living child is achieved. Exclusion criteria were previous surgical destruction of superficial endometriotic lesions, as these women may have wrongly been misclassified on ultrasound examination as not having endometriosis, and current hormonal treatment, as this may alter the sonographic appearance of the myometrium. Superficial peritoneal endometriosis, only visible by laparoscopy, was not assessed for. Oocyte donation cycles were not included.

### Ultrasound examination

All women filled in a questionnaire regarding symptoms and underwent a systematic transvaginal 2- (2D) and 3-dimensional (3D) ultrasound examination conducted by the first author before starting treatment, as previously described (Alson *et al.*, 2022, 2024a,b).

The pelvis was assessed for the presence of DE and the uterus for features of adenomyosis. Endometriomas were defined as unilocular cysts with ground glass echogenicity (Van Holsbeke *et al.*, 2010). DE was described using the IDEA terminology (Guerriero *et al.*, 2016). Adenomyosis was diagnosed based on the revised MUSA definitions (Harmsen *et al.*, 2022). According to these, direct features (subendometrial lines and buds, hyperechogenic islands, and myometrial cysts) are pathognomonic of adenomyosis, whereas indirect features (irregular or interrupted junctional zone, myometrial asymmetry, globular shape of the uterus, fan-shaped shadowing, or translesional vascularity) are only indicative of the disease (Harmsen *et al.*, 2022). Women with only indirect features, but without direct features, were therefore not classified as having adenomyosis. The number and extent of features within the myometrium were determined, as previously described (Alson *et al.*, 2024a,b). Adenomyosis was classified as focal, diffuse, or mixed-type, and described as being localized in the inner, middle, or outer myometrium (Van den Bosch *et al.*, 2019; Alson *et al.*, 2024a,b). Additionally, the antral follicle count (AFC) was defined as the sum of all follicles 2–10 mm in both ovaries.

### Patient treatment

Serum-anti-Müllerian hormone was analyzed before the first treatment. All consecutive IVF/ICSI treatments took place between January 2019 and April 2024. The first treatment cycle has been previously described in detail (Alson *et al.*, 2024a,b), and subsequent treatments were adjusted based on individual patient characteristics or preferences and response to prior treatments. If fewer than three follicles matured, the cycle was either canceled or converted to IUI and therefore not included in the final analysis.

Embryos were graded using the Gardner blastocyst grading scale (Gardner and Schoolcraft, 1999). Embryo transfer was performed either at the cleavage stage (Days 2–3) or at the blastocyst stage (Day 5), with single embryo transfer as standard practice. Surplus good-quality embryos (GQEs) or embryos from patients unable to undergo fresh ET were cryopreserved on Days 5–6 post-oocyte pickup (OPU). FET was performed in natural or hormone replacement cycles. Luteal phase support was provided for two weeks after OPU using progesterone vaginal suppositories (Lutinus, Ferring, Lausanne, Switzerland). Embryos from each treatment cycle were used until a live birth (LB) (≥22 gestational weeks) was achieved or no embryos remained. If LB was not achieved after the first cycle, women were offered up to two additional IVF/ICSI cycles.

### Primary outcome

The primary outcome was CLBR after three consecutive IVF/ICSI cycles, including both fresh and frozen ETs, in women with or without E/A, as defined by the IDEA (Guerriero *et al.*, 2016) and revised MUSA criteria (Harmsen *et al.*, 2022).

### Secondary outcome

Secondary outcomes were LBR after each IVF/ICSI cycle, stratified for fresh or frozen transfers as well as for women with only endometriosis, only adenomyosis, or with concurrent conditions.

### Power analysis

A previous publication has suggested that women with E/A have a pregnancy rate of 31% after IVF-FET compared with 52.9% in the control group (Wang *et al.*, 2023). To achieve 80% power with a two-sided alpha level of 0.05, a total of 76 women with E/A were required to detect a statistically significant difference between the two groups.

### Statistical analyses

Statistical analyses were performed using IBM SPSS Statistics for Windows, Version 29.0 (IBM Corp., released 2020, Armonk, NY, USA).

Normally distributed data are presented as means with standard deviations (±SD), and non-normally distributed data as medians with ranges. Proportions are expressed as percentages. To compare proportions between groups, the chi-square test or Fisher’s exact test was used. The Student’s *t*-test was used for comparing normally distributed data, while the Mann–Whitney *U*-test was used to compare median values between groups. A *P*-value <0.05 was considered statistically significant.

To estimate the relative risk (RR) for CLB in women with E/A, a modified Poisson regression with robust error variances was performed, before and after adjusting for potential confounders. To identify potential confounders in the association between E/A and CLBR, a directed acyclic graph (DAG) was constructed, and a sensitivity analysis was performed. A variable was considered a confounder if its inclusion changed the RR of the main exposure by >10%. The variables tested were AMH, age, AFC, BMI, embryo stage, ART protocol, fertilization method, and FET. Variables that were identified as potential confounders in the DAG were included even if they did not change the RR estimate by >10%, to avoid confounding bias. RR is presented with 95% CIs.

The primary analysis was conducted on an intention-to-treat (ITT) basis, including all women who initiated IVF treatment regardless of whether they completed all three cycles. To reflect treatment efficacy among those who completed all cycles, a secondary per-protocol (PP) analysis was performed by excluding women who discontinued treatment before completing all three cycles. A Kaplan–Meier analysis was performed to help account for dropouts (censored data) and to estimate the probability of LB over three cycles. A log-rank test was used to estimate differences between groups.

A subgroup analysis was performed for women with different disease phenotypes. Results for women with indirect features of adenomyosis are presented as supplementary data.

### Ethics

This study was approved by the Regional Ethical Review Board of Lund University, Lund, Sweden, on 11 September 2018, with a reference number 2018/555. Informed, written consent was obtained from all participants. The study was conducted according to the Declaration of Helsinki for Medical Research.

## Results

**Figure 1.**
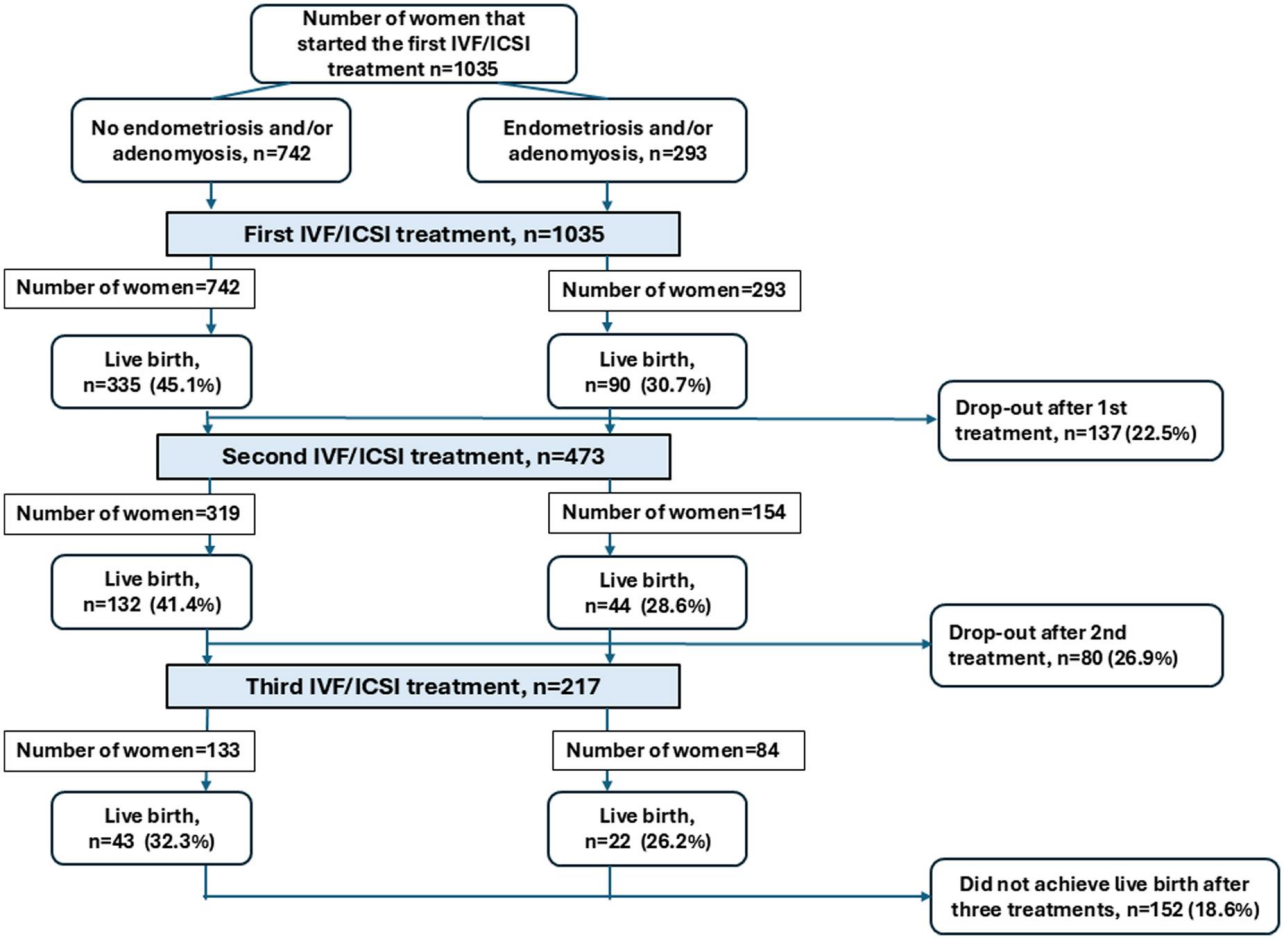
Flowchart demonstrating the number of women undergoing each IVF/ICSI treatment cycle, live births, and drop-outs. n, number.

All 1035 women underwent the first IVF/ICSI cycle. Of the 610/1035 (58.9%) women who did not achieve LB after the first cycle, 473/610 (77.5%) women proceeded with the second treatment. Of the 297/473 (62.8%) women that thereafter remained, 217/297 (73.1%) women underwent the third treatment (Fig. 1). In total, 818 (79.0%) women underwent all treatments they were eligible for. Of these, 593 (72.7%) women did not have E/A, whereas 223 (27.3%) women had ultrasonographic signs of either or both diseases. The reasons why the remaining 217/1035 (21.0%) women dropped out after one or two treatments are presented in Supplementary Table S1.

The background characteristics of the women included in the study are presented in Table 1. The distribution of typical symptoms is presented in Supplementary Table S2.

**Table 1.** Background characteristics of the women included in the study, and for women with versus without endometriosis and/or adenomyosis.

Parameter	Total cohort, n = 1035	No endometriosis and/or adenomyosis n = 742	Endometriosis and/or adenomyosis, n = 293
Age, years	32.0 ± 4.0	31.7 ± 3.9	32.7 ± 4.1
BMI, kg/m^2^	23.9 ± 3.4	23.9 ± 3.4	23.9 ± 3.5
Menstrual cycle length, days	28 (18–90)	28 (21–90)	28 (18–90)
s-AMH, pmol/L	18 (0.2–244)	20 (0.3–244)	16 (0.2–87)
AFC	17 (0–90)	18 (0–90)	14 (2–50)
Years of subfertility	2.5 ± 2	2.5 ± 2	2.5 ± 2
Main indication for ART			
Unexplained	367 (35.5)	338 (45.6)	29 (9.9)
Male	329 (31.8)	256 (34.5)	73 (24.9)
Mixed^a^	35 (3.4)	24 (3.2)	11 (3.8)
Tubal	78 (7.5)	55 (7.4)	23 (7.8)
Endometriosis	143 (13.8)	0 (0)	143 (48.8)
Oligo-/amenorrhea^b^	62 (6.0)	55 (7.4)	7 (2.4)
Other^c^	21 (2.0)	14 (1.9)	7 (2.4)
Previous childbirth,	43 (4.2)	27 (3.6)	16 (5.5)
Previous termination of pregnancy	108 (10.4)	78 (10.5)	30 (10.2)
Previous miscarriage	138 (13.3)	97 (13.1)	41 (14.0)
Myoma	158 (15.3)	113 (15.2)	45 (15.4)

s-AMH, serum anti-Müllerian hormone; AFC, antral follicle count; TVUS, transvaginal ultrasonography.

a Mixed=male and female factors.

b Including women with polycystic ovarian syndrome.

c Other=same gender couples or single woman. Values are given as n (%) of each group of women, mean (±SD), or median (range).

A total of 232 (22.4%) women had endometriosis; 114 (11.0%) had endometriomas (with or without DE), and 183 (17.7%) had DE (with or without endometriomas). Direct features of adenomyosis were present in 102 (9.9%) women; 52 (5.0%) had hyperechogenic islands, 36 (3.5%) had myometrial cysts, and 76 (7.3%) women had subendometrial lines and buds. In total, 293 (28.3%) women had either endometriosis and/or direct features of adenomyosis. Of these, 41 (4.0%) women had concurrent endometriosis and adenomyosis, 191 (18.5%) women had endometriosis only, and 61 (5.9%) women had only adenomyosis. The adenomyosis phenotype and number of features are presented in Supplementary Table S3. In total, 40 (3.9%) women had two or more direct features, and 161 (15.6%) women had two or more direct and/or indirect features of adenomyosis.

The IVF/ICSI treatment methods and outcomes in three consecutive cycles are presented in Tables 2 and 3, respectively.

**Table 2.** IVF/ICSI treatment in three consecutive cycles for women without or with endometriosis and/or adenomyosis.

Parameter	Total cohort, n = 1035	No endo/adeno, n = 742	Endo and/or adeno, n = 293	*P*-value
**First cycle, n = 1035**	**N = 1035**	**N = 742**	**N = 293**	
GnRH downregulation	27 (2.6)	3 (0.4)	24 (8.2)	<0.001*
**Protocol**				
Agonist	139 (13.4)	57 (7.7)	82 (28.0)	<0.001*
Antagonist	896 (86.6)	685 (92.3)	211 (72.0)	<0.001*
Nb of stimulation days	11 (2–19)	10 (2–19)	11 (9–17)	0.044*
FSH dose (IU)	1775 (217–5325)	1725 (217–3500)	2000 (1425–5325)	<0.001*
Nb of mature oocytes	7 (2–20)	7 (2–19)	7 (3–20)	0.020*
**Second cycle, n = 473**	**N = 473**	**N = 319**	**N = 154**	
GnRH downregulation	6 (1.3)	2 (0.6)	4 (2.6)	0.077
**Protocol**				
Agonist	181 (38.3)	111 (34.8)	70 (45.5)	0.027*
Antagonist	292 (61.7)	208 (65.2)	84 (54.5)	0.027*
Nb of stimulation days	11 (9–16)	11 (9–16)	12 (9–15)	0.109
FSH dose (IU)	2332 (200–7200)	2250 (200–4000)	2850 (1700–7200)	0.057
Nb of mature oocytes	6 (0–20)	8 (0–20)	6 (1–14)	0.570
**Third cycle, n = 217**	**N = 217**	**N = 133**	**N = 84**	
GnRH downregulation	10 (4.6)	1 (0.8)	9 (10.7)	0.003*
**Protocol**				
Agonist	139 (64.1)	78 (58.6)	61 (72.6)	0.042*
Antagonist	78 (35.9)	55 (41.4)	23 (27.4)	0.042*
Nb of stimulation days	12 (8–16)	12 (9–16)	11 (8–15)	0.620
FSH dose (IU)	2550 (500–6300)	2587 (1000–6300)	2137 (500–5500)	0.249
Nb of mature oocytes	6 (1–27)	9 (1–27)	5.5 (1–18)	0.336

Adeno, adenomyosis; endo, endometriosis; IU, international unit.

Values are given as n (%) of each group of women or median (range).

* *P* < 0.05, assessed by chi-square test or Mann–Whitney *U*-test as appropriate, is considered to indicate a statistically significant difference between groups.

**Table 3.** IVF/ICSI treatment and outcome in three consecutive cycles.

Parameter	Total cohort, n = 1035	No endo/adeno, n = 742	Endo and/or adeno, n = 293	*P*-value
**First cycle, n = 1035**	**N = 1035**	**N = 742**	**N = 293**	
ART method^a^, n (%)				
IVF	583 (56.3)	396 (53.4)	187 (63.8)	<0.001*
ICSI	403 (38.9)	314 (42.3)	89 (30.4)	<0.001*
Fertilization rate	0.52 ± 0.29	0.52 ± 0.28	0.52 ± 0.29	0.96
Nb of GQE	1 (0–3)	1 (0–4)	1 (0–3)	0.27
ET stage^b^, n (%)	**N = 760**	**N = 546**	**N = 214**	
Cleavage stage	395 (52.0)	267 (48.9)	128 (59.8)	0.006*
Blastocyst stage	365 (48.0)	279 (51.1)	86 (40.2)	0.015*
Women with all GQE frozen	61 (5.9)	47 (6.3)	14 (4.8)	0.653
Nb of FET cycles	509	335	174	
**Second cycle, n = 473**	**N = 473**	**N = 319**	**N = 154**	
ART method^a^				
IVF	159 (33.6)	92 (28.8)	67 (43.5)	<0.001*
ICSI	297 (62.8)	214 (67.1)	83 (53.9)	0.002*
Fertilization rate	0.54 ± 0.48	0.57 ± 0.55	0.49 ± 0.31	0.037*
Nb of GQE	1 (0–3)	1 (0–3)	1 (0–3)	0.107
ET stage^b^	**N = 367**	**N = 245**	**N = 122**	
Cleavage stage	237 (64.6)	151 (61.6)	86 (70.5)	0.107
Blastocyst stage	130 (35.4)	94 (38.4)	36 (29.5)	0.107
Women with all GQE frozen	27 (5.7)	19 (6.0)	8 (5.2)	0.834
Nb of FET cycles	222	146	76	
**Third cycle, n = 217**	**N = 217**	**N = 133**	**N = 84**	
ART method^a^				
IVF	53 (24.4)	29 (21.8)	24 (28.6)	0.255
ICSI	155 (71.4)	99 (74.4)	56 (66.7)	0.255
Fertilization rate	0.57 ± 0.26	0.55 ± 0.26	0.61 ± 0.27	0.185
Nb of GQE	2 (0–5)	2 (0–5)	1 (0–3)	0.826
ET stage^b^	**N = 156**	**N = 96**	**N = 60**	
Cleavage stage	130 (83.3)	77 (80.2)	53 (88.3)	0.190
Blastocyst stage	26 (16.7)	19 (19.8)	7 (11.7)	0.190
Women with all GQE frozen	11 (5.1)	6 (4.5)	5 (6.0)	0.752
Nb of FET cycles	103	58	45	

Adeno, adenomyosis; endo, endometriosis; ET, embryo transfer; FET, frozen embryo transfer; GQE, good-quality embryo.

a For the remaining women, fertilization was not possible due to lack of mature oocytes or sperm.

b Some women had all embryos frozen or did not achieve any GQE, why percentages are calculated per freshly transferred embryos. Values are given as n (%) of each group of women, mean (±SD), or median (range).

* *P* < 0.05, assessed by chi-square test, Student’s *t*-test, or Mann–Whitney *U*-test as appropriate, is considered to indicate a statistically significant difference between groups.

**Figure 2.**
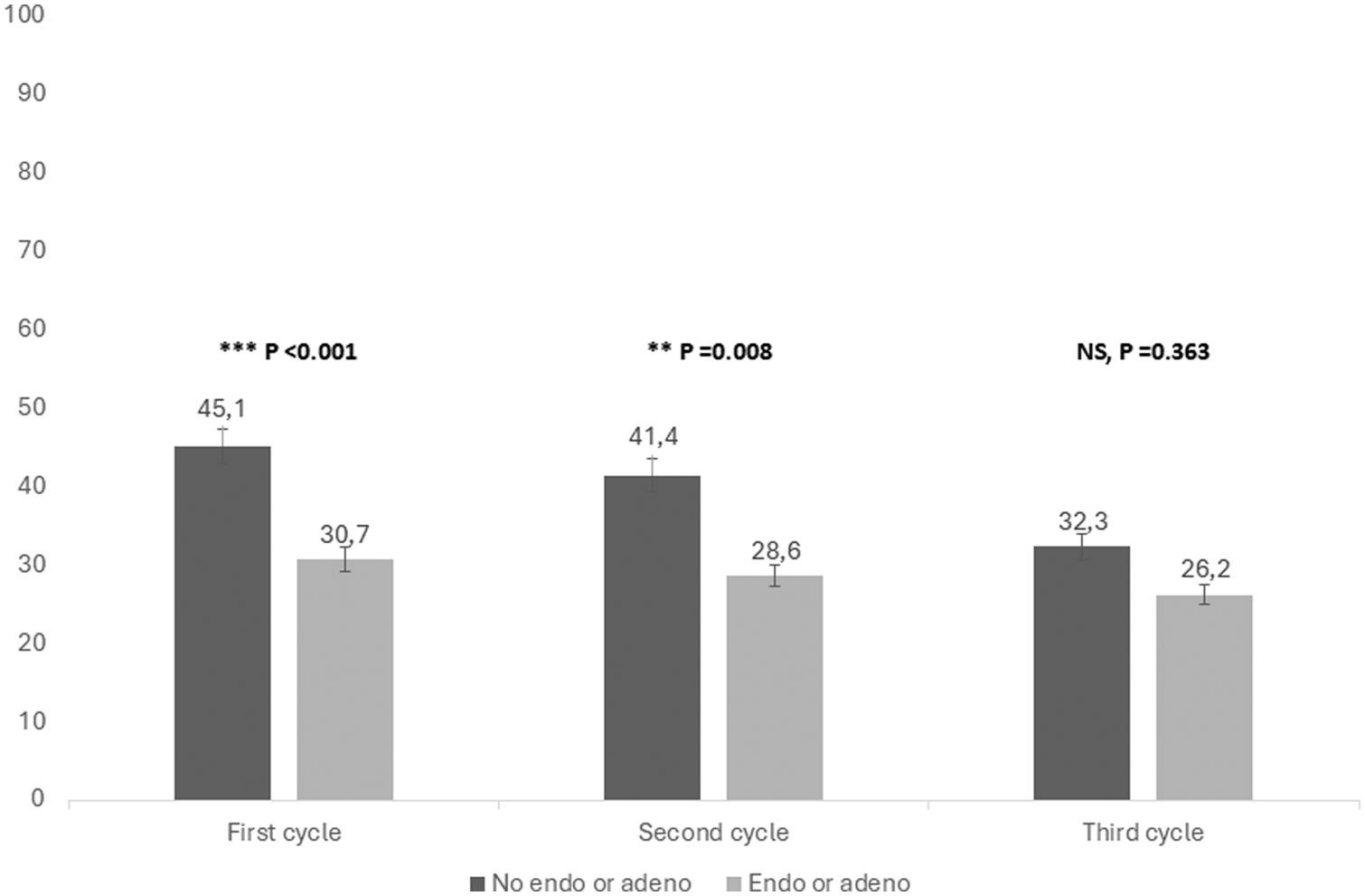
**Live birth rates in three consecutive IVF/ICSI treatment cycles, for women with and without endometriosis and/or adenomyosis.** Endo, endometriosis; adeno, adenomyosis. Numbers are given in percentages (SD). *P*<0.05 is considered to indicate a statistically significant difference.

**Figure 3.**
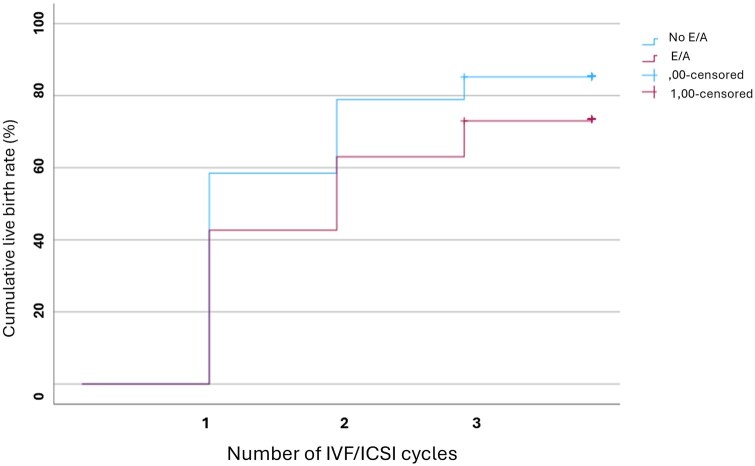
**Observed cumulative live birth rate in three consecutive IVF/ICSI cycles.** No E/A, women without endometriosis and/or adenomyosis; E/A, women with endometriosis and/or adenomyosis; ,00 censored, women without E/A who had not achieved a live birth by the end of observation or who discontinued treatment before completing three IVF/ICSI cycles; 1,00 censored, women with E/A who had not achieved a live birth by the end of observation or who discontinued treatment before completing three IVF/ICSI cycles. The number of IVF/ICSI cycles is one to three.

In total, 1725 fresh cycles were initiated, and 1283 fresh and 831 frozen ETs were performed. A total of five cycles were converted to IUI and therefore excluded. The average number of FET per woman was similar between the two groups (0.45 FET/woman without E/A and 0.55 FET/woman with E/A, *P* = 0.220). The CLBR for the total cohort after three consecutive IVF/ICSI treatment cycles was, calculated for women who initiated treatment (ITT), 666/1035 (64.3%, 95% CI; 60.2–66.0), or 666/818 (81.4%; 95% CI 78.9–84.3) for women who completed all cycles they were eligible for (PP), Table 4. In the ITT and PP analyses, respectively, the CLBR for women without E/A was 510/742 (68.7%, 95% CI, 65.4–72.0) or 510/595 (85.7%, 95% CI 83.2–88.8), compared to 156/293 (53.2%, 95% CI 47.3–58.7) or 156/223 (70.0%, 95% CI 64.0–76.0) for women with the diseases, *P* < 0.001, Table 4. The adjusted RR (aRR) for LB for women with E/A was, in the ITT analysis, 0.80 (95% CI, 0.71–0.90), and in the PP analysis, 0.85 (95% CI, 0.77–0.93), *P* < 0.001. The LBR for each of the three consecutive IVF/ICSI cycles is presented in Table 4. The LBR for women with E/A remained relatively stable across the three consecutive treatment cycles, Fig. 2. The mean number of treatments to LB for women with or without E/A is presented in Table 5 and Fig. 3. The LBR after fresh or frozen ET in respective IVF/ICSI cycle, stratified for women with different disease phenotypes, is presented in Table 6. The difference in LBR was mainly attributed to differences in LBR after fresh transfers. The CLBR for women with ≥2 direct features was lower than for healthy women (28/40 (70.0%), *P* = 0.013). When stratifying endometriosis by disease severity, the presence of DE did not impact CLBR significantly compared to healthy women (PP; 67/87, 77.0%, vs 534/638, 83.7%, *P* = 0.120), whereas endometriomas were associated with a reduced CLBR (PP; 25/37, 67.6% vs 534/638, 83.7%, *P* = 0.011). Women with DE and endometrioma had a PP CLBR of 38/53 (71.7%) compared to 534/638 (83.7%) for women without, *P* = 0.026.

**Table 4.** IVF/ICSI treatment outcomes per cycle, and cumulative outcomes after three consecutive cycles.

Parameter, n (%)	Total cohort, n = 1035 N (%)	No endo/adeno, n = 742 N (%)	Endo and/or adeno, n = 293 N (%)	Crude RR	95% CI	*P*-value	**Adjusted** ^a^ **RR**	95% CI	*P*-value
**First cycle, n = 1035**	**N = 1035**	**N = 742**	**N = 293**						
Pregnancy rate	528 (51.0)	416 (56.1)	112 (38.2)	0.76	0.65–0.88	<0.001*	0.77	0.66–0.91	0.001*
Miscarriage rate	103 (10.0)	81 (10.9)	22 (7.5)	1.26	1.02–1.55	0.033*	1.13	1.04–1.23	0.003*
Live birth rate	425 (41.1)	335 (45.1)	90 (30.7)	0.68	0.56–0.82	<0.001*	0.69	0.57–0.84	<0.001*
**Second cycle, n = 473**	**N = 473**	**N = 319**	**N = 154**						
Pregnancy rate	221 (46.7)	161 (50.5)	60 (39.0)	0.79	0.62–1.02	0.076	0.82	0.64–1.06	0.136
Miscarriage rate	45 (9.5)	29 (9.1)	16 (10.4)	1.077	0.60–1.93	0.802	1.06	0.59–1.90	0.847
Live birth rate	176 (37.2)	132 (41.4)	44 (28.6)	0.69	0.52–0.91	0.009*	0.72	0.54–0.96	0.023*
**Third cycle, n = 217**	**N = 217**	**N = 133**	**N = 84**						
Pregnancy rate	86 (39.6)	58 (43.6)	28 (33.3)	0.80	0.55–1.16	0.241	0.82	0.56–1.20	0.306
Miscarriage rate	22 (10.1)	15 (11.3)	7 (8.3)	1.83	0.34–9.92	0.482	1.47	0.54–4.04	0.455
Live birth rate	65 (30.0)	43 (32.3)	22 (26.2)	0.81	0.52–1.25	0.343	0.83	0.54–1.28	0.183
**Cumulative results for three IVF/ICSI treatment cycles**	**N = 818**	**N = 595**	**N = 223**						
Cumulative PR^b^	835 (80.7)	635 (85.6)	200 (68.3)	0.88	0.82–0.95	0.001*	0.89	0.83–0.96	0.003*
Cumulative pregnancy loss^b^	169 (16.3)	125 (16.8)	44 (15.0)	0.71	0.50–1.03	0.069	0.74	0.51–1.06	0.101
CLBR after three IVF/ICSI treatment cycles (ITT)^c^	666 (64.3)	510 (68.7)	156 (53.2)	0.78	0.69–0.88	<0.001*	0.80	0.71–0.90	<0.001*
CLBR after three IVF/ICSI treatment cycles (PP)^d^	666 (81.4)	510 (85.7)	156 (70.0)	0.84	0.76–0.92	<0.001*	0.85	0.77–0.93	<0.001*

Adeno, adenomyosis; endo, endometriosis; ITT, intention to treat; PP, per-protocol analysis; RR, relative risk; CLBR, cumulative live birth rate.

a Adjusted for age.

b Calculated per woman who started treatment.

c Calculated for all women who initiated the first treatment cycle (ITT).

d Calculated for women who completed all cycles they were eligible for (PP). Values are given as n (%).

* *P* < 0.05 is considered to indicate a statistically significant difference between groups.

**Table 5.** Mean number of IVF/ICSI treatments to live birth for women with or without endometriosis and/or adenomyosis.

Disease status	Mean nb of IVF/ICSI treatments	95% CI	Log-rank *P*-value
Total cohort, n = 1035	1.89	1.81–1.97	
No endo and/or adeno, n = 742	1.78	1.69–1.86	
Endo and/or adeno, n = 293	2.21	2.04–2.38	<0.001*

Adeno, adenomyosis; endo, endometriosis.

* *P* < 0.05 is considered to indicate statistically significant difference.

**Table 6.** IVF/ICSI treatment outcomes per cycle, stratified per disease phenotype, and type of embryo transfer.

Parameter, n (%)	Total cohort, n = 1035	No endo/adeno, n = 742	Endo and/or adeno, n = 293	*P*-value	**Endo only** ^a^ **, n = 191**	***P*-value** ^c^	**Adeno only** ^b^ **, n = 61**	***P*-value** ^c^	**Endo and Adeno** ^d^ **n = 41**	***P*-value** ^c^
**First cycle, n = 1035**	**N = 1035**	**N = 742**	**N = 293**		**N = 191**		**N = 61**		**N = 41**	
Live birth fresh	295 (38.8)	237 (32.0)	58 (19.8)	<0.001*	40 (20.9)	0.003*	9 (15.0)	0.006*	9 (22.0)	0.381
Live birth frozen	130 (43.8)	98 (46.0)	32 (38.0)	0.243	25 (43.9)	0.881	5 (29.4)	0.185	2 (20.0)	0.195
Total Live birth rate	425 (41.1)	335 (45.1)	90 (30.7)	<0.001*	65 (34.0)	0.006*	14 (23.0)	<0.001*	11 (26.8)	0.021*
**Second cycle, n = 473**	**N = 473**	**N = 319**	**N = 154**		**N = 97**		**N = 35**		**N = 22**	
Live birth fresh	118 (32.2)	87 (41.4)	31 (22.0)	<0.001*	23 (23.7)	0.003*	4 (17.4)	0.026*	4 (18.2)	<0.001*
Live birth frozen	58 (46.8)	45 (52.9)	13 (33.3)	0.042*	9 (32.0)	0.136	1 (25.0)	0.350	3 (30.0)	0.334
Total Live birth rate	176 (37.2)	132 (41.4)	44 (28.6)	0.006*	32 (32.0)	0.133	5 (14.3)	0.002*	7 (46.7)	0.371
**Third cycle, n = 217**	**N = 217**	**N = 133**	**N = 84**		**N = 52**		**N = 22**		**N = 10**	
Live birth fresh	44 (28.2)	28 (21.2)	16 (19.2)	0.834	11 (23.9)	0.964	5 (22.7)	0.781	0	0.362
Live birth frozen	21 (20.4)	15 (45.5)	6 (31.6)	0.511	2 (20.0)	0.359	2 (40.0)	0.785	2 (50.0)	0.791
Total Live birth rate	65 (30.0)	43 (32.3)	22 (26.2)	0.310	13 (28.3)	0.264	7 (31.8)	0.962	2 (22.2)	0.528
**Cumulative LBR (ITT)**	666 (64.3)	510 (68.7)	156 (53.2)	<0.001*	110 (57.6)	<0.001*	26 (42.6)	<0.001*	20 (48.8)	<0.001*
**Cumulative LBR (PP)**	666 (81.4)	510 (85.7)	156 (70.0)	<0.001*	110 (33.3)	<0.001*	26 (22.0)	<0.001*	20 (27.8)	<0.001*

Adeno, adenomyosis; endo, endometriosis; ITT, intention-to-treat analysis; LBR, live birth rate; live birth fresh, live birth after fresh embryo transfer; live birth frozen, live birth after frozen thawed embryo transfer; PP, per-protocol analysis.

a Endometriosis with direct features excluded.

b Direct features without endometriosis.

c The *P*-value was counted for the difference between women with the disease phenotype and women without any disease. chi-square test, or Fisher’s exact test when the expected cell count was low, was used to compare proportions between groups.

d Endometriosis and direct features of adenomyosis. Live birth rates are calculated per woman with fresh or frozen transfer in each cycle. Data are presented as numbers (%).

* *P*-values <0.05 are indicating statistically significant difference.

For women with E/A, comparison of background characteristics, disease severity and treatments between those who did or did not achieve LB is presented in Supplementary Table S4.

## Discussion

In this study, we found that women with E/A had a 15% lower cumulative chance of achieving an LB after three consecutive IVF/ICSI cycles compared to women without these conditions. The most pronounced difference was observed in the first cycle (aRR for LB 0.69), with a persistent but smaller gap in the second cycle (aRR 0.72), and a non-significant difference in the third cycle (aRR 0.83, *P* = 0.183). The largest disparities were seen after fresh ET. Our findings highlight the detrimental impact of endometriosis and adenomyosis on IVF/ICSI outcomes, particularly in the initial treatment cycles, but suggest that repeated attempts may help mitigate some of the initial disadvantages associated with these conditions.

Our findings align with previous studies reporting reduced IVF success in women with either condition (Mavrelos *et al.*, 2017; Muteshi *et al.*, 2018), often attributed to impaired implantation, inflammation, or altered uterine function. Studies focusing on endometriosis alone have reported lower implantation rates, increased miscarriage rates, and reduced CLBR, particularly in women with advanced disease (Kuivasaari *et al.*, 2005). Adenomyosis has been associated with reduced endometrial receptivity and a 28% lower chance of clinical pregnancy (Vercellini *et al.*, 2014).

Although fewer studies have addressed the combined impact of both diseases, recent evidence supports a compounded negative effect with reduced CLBR (Wang *et al.*, 2023). For example, Ballester *et al.* (2012) and Bourdon *et al.* (2022) found significantly lower cumulative pregnancy and LBR in women with both diagnoses. Our results support these findings and demonstrate that the negative impact is most pronounced early in treatment but may be mitigated with repeated cycles and individualized care.

Contradictory findings in other studies (Gonzalez-Comadran *et al.*, 2017; Higgins *et al.*, 2021; Somigliana *et al.*, 2023) may stem from differences in diagnostic criteria and methods, study design, and ART protocols. Many studies are retrospective, rely on surgical or varied imaging diagnostics, and often only examine outcomes from the first cycle. (Horton *et al.*, 2019). In contrast, we used prospective data with standardized ultrasound-based criteria and followed patients across three consecutive IVF/ICSI attempts. Analyzing outcomes over multiple cycles reflects clinical reality more accurately than first-cycle-only studies. Protocols often change between cycles based on prior results, potentially improving outcomes over time. For example, in our study, women with E/A increasingly received the agonist protocol in later cycles, a strategy linked to higher pregnancy rates in endometriosis (Liu *et al.*, 2021). Similarly, more couples underwent ICSI in subsequent cycles, potentially mitigating undetected male factor infertility. These changes may explain why the LB gap between groups narrowed by the third cycle. If a correct diagnosis is made before initiating the first treatment, this could be planned accordingly and potentially lead to a higher success rate even in the first cycles (Alsbjerg *et al.*, 2023; Humaidan *et al.*, 2023; Cozzolino *et al*., 2024a).

Notably, the LBR for women with E/A remained relatively stable across three cycles (30.7%, 28.6%, 26.2%), whereas it declined in women without these conditions (from 45.1% to 32.3%). Several factors may explain this convergence. Firstly, untreated adenomyosis may result in persistent uterine dysfunction (Brosens *et al.*, 2010; Bulun *et al.*, 2023), while protocol adaptations (e.g. agonist protocol, ICSI) may have enhanced outcomes in later cycles. Secondly, selection bias likely played a role. Women without E/A who conceived early exited the study, leaving a cohort with potentially poorer prognosis. This evolving population composition likely reduced observed differences by the third cycle.

To address selection bias and to reflect treatment efficacy, we presented results on both an ITT and PP basis. Dropout is common in IVF (Smith *et al.*, 2015; Li *et al.*, 2018), driven by factors such as relationship issues, financial concerns, or clinical discouragement (Gameiro *et al.*, 2012). Our use of Kaplan–Meier analyses and adjusted risk ratios with and without dropouts helped mitigate this limitation.

A strength of our study is the prospective design, clear diagnostic criteria for endometriosis and adenomyosis, and a well-defined consecutive population. All ultrasound examinations were undertaken by an examiner with expertise in ultrasonography using 2D/3D TVUS, according to the internationally accepted IDEA and revised MUSA criteria, ensuring diagnostic uniformity and accurate diagnoses even for women without typical symptoms suggestive of these diseases. Using direct features to diagnose adenomyosis is a strength, as these have high specificity for adenomyosis in comparison with histology (Harmsen *et al.*, 2022). However, this approach may limit generalizability to clinics without similar expertise or equipment.

A potential limitation of this study is the lack of laparoscopic or histopathologic confirmation. Although the IDEA method reliably detects DE compared with laparoscopy (Goncalves *et al.*, 2021; Leonardi *et al.*, 2022), superficial lesions may have gone undetected, leading to potential misclassification and bias. However, laparoscopy is not part of standard infertility work-up (Becker *et al.*, 2022), and hysterectomy is not appropriate in women desiring pregnancy. Surgical confirmation would not have been feasible nor ethically justified, an inherent limitation shared by similar studies. Even if significantly more women with E/A had typical symptoms, a large proportion of the control group also reported symptoms, some of which may have had undetected disease.

Similarly, some cases of adenomyosis may have been misclassified. Although indirect features are not diagnostic in the absence of direct signs (Harmsen *et al.*, 2022), they may nonetheless indicate disease in some cases. Moreover, the reproductive impact of asymptomatic adenomyosis remains debated (Neal *et al.*, 2020) and was not accounted for in this study. While some experts use a threshold of ≥2 direct or indirect features to enhance specificity and reduce overdiagnosis (Bourdon *et al.*, 2024), we adhered to the MUSA definition to ensure high specificity while capturing early-stage or mild cases with possible reproductive significance (Cozzolino *et al.*, 2024b). Consequently, some women without disease could wrongly have been classified has having adenomyosis. The number of features necessary for a diagnosis should be discussed in a future revision of the MUSA definitions.

We also acknowledge the simplification of treating endometriosis and adenomyosis as binary variables, as it does not reflect phenotypic variation or potential dose–response effects on reproductive outcomes. Disease presence and severity may have been under- or overestimated in the absence of MRI or histological confirmation. Moreover, internal and external adenomyosis may have different clinical profiles and thus different impacts on fertility (Bourdon *et al.*, 2021; Alson *et al.*, 2024a,b; Valdes-Bango *et al.*, 2024). While no internationally accepted ultrasonographic classification exists for severity, unlike the rASRM surgical staging system for endometriosis (Practice Committee of the American Society for Reproductive Medicine, 2012), most IVF protocols base management on disease presence rather than stage. Still, this ‘all-or-nothing’ approach may introduce overdiagnosis and obscure phenotype-specific effects. To address this, we conducted subgroup analyses by disease phenotype which showed consistent results despite limited power. Neither endometriosis (DE or endometrioma) nor adenomyosis phenotypes (internal, external, diffuse, focal, myometrial layer) were significantly different between women with E/A who did or did not achieve LB.

Unlike studies that excluded embryonic factors by using only blastocysts (Bourdon *et al.*, 2022, 2024), we included both cleavage- and blastocyst-stage embryos, as well as fresh and FET cycles. This approach may be criticized, as several studies have reported similar ART outcomes for women with or without endometriosis or adenomyosis after blastocyst FET (Neal *et al.*, 2020; Bishop *et al.*, 2021). In line with their results, the largest differences in LBR in our study were observed after fresh ET. Although these numbers must be interpreted with caution due to small subgroup numbers, they strengthen the hypothesis that in FET cycles, the endometrium is not exposed to the supraphysiologic estrogen levels as in fresh transfers, why FET could be preferable for women with E/A (Neal *et al.*, 2020; Bishop *et al.*, 2021). Importantly, we used individualized protocols believed to offer the highest success chance, minimizing selection bias that could overestimate CLBR if women with failed blastocyst development or canceled stimulations were excluded. The proportion of women undergoing blastocyst transfer or FET was similar across groups in subsequent treatment cycles. Our decision to include both fresh and FET cycles, using individualized treatment strategies, aligns with clinical practice. It would neither have been ethically nor medically justified to use the same treatment protocol for all women merely for study purposes. This may limit our ability to determine whether specific protocols (e.g. deferred FET, downregulation before FET or exogenous progesterone supplementation) benefit women with E/A. Previous studies suggest protocol-specific advantages (Alsbjerg *et al.*, 2023; Cozzolino *et al.*, 2024a), but these remain to be confirmed.

Finally, concerns have been raised about embryo quality in women with endometriosis, though recent studies show no significant difference in aneuploidy rates compared to unaffected IVF patients (Juneau *et al.*, 2017). Our study did not include embryo genetic testing, which could further clarify this issue.

In conclusion, our results show that women with E/A have a reduced chance of having an LB after three consecutive IVF/ICSI treatments compared to women without these conditions. However, the chance of having a child does not necessarily decline with additional, modified, treatments. Negative results after the first treatment should not be a reason to withhold further attempts. Future research should explore strategies to enhance treatment success in this population, including the role of long-term suppression protocols, exogenous progesterone dosing, personalized embryo transfer approaches, and more targeted interventions for endometriosis or adenomyosis-related implantation failure.

## Supplementary Material

deaf184_Supplementary_Table_S1

deaf184_Supplementary_Table_S2

deaf184_Supplementary_Table_S3

deaf184_Supplementary_Table_S4

## Data Availability

The data underlying this article cannot be shared publicly due to ethical reasons and for the privacy of the participants. The data will be shared at reasonable request to the corresponding author.
