# Supplementary material for: Cumulative live birth rates under three consecutive IVF/ICSI treatment cycles are reduced in women with endometriosis and/or adenomyosis diagnosed by ultrasonography
Source: Hum Reprod. 2025 Sep 20;40(12):2332–41. doi: 10.1093/humrep/deaf184 (PMC12675419; doi:10.1093/humrep/deaf184)
Supplement: deaf184_Supplementary_Table_S1 [file deaf184_supplementary_table_s1.pdf]

OXFORD
human reproduction

Human Reproduction, 2025, 1–1
https://doi.org/10.1093/humrep/deaf184
Supplementary Data

Supplementary Table S1. Reason for not undergoing all eligible treatments.

Reason for drop-out	Total cohort, n = 217	Women without endo and/or adeno, n = 147	Women with endo and/or adeno, n = 70
Spontaneous pregnancy	16 (7.4)	14 (9.5)	2 (2.9)
Personal issues	30 (13.8)	15 (10.2)	15 (21.4)
Severe health problem	10 (4.6)	8 (5.4)	2 (2.9)
Age ≥40 years	32 (14.7)	14 (9.5)	18 (25.7)
Awaiting oocyte donation	21 (9.7)	11 (7.5)	10 (14.3)
Unknown reason	108 (49.8)	85 (57.8)	23 (32.9)

Endo, endometriosis; adeno, adenomyosis. Numbers are given as n (%).
