# Supplementary material for: Cumulative live birth rates under three consecutive IVF/ICSI treatment cycles are reduced in women with endometriosis and/or adenomyosis diagnosed by ultrasonography
Source: Hum Reprod. 2025 Sep 20;40(12):2332–41. doi: 10.1093/humrep/deaf184 (PMC12675419; doi:10.1093/humrep/deaf184)
Supplement: deaf184_Supplementary_Table_S2 [file deaf184_supplementary_table_s2.pdf]

Human Reproduction, 2025, 1–1
https://doi.org/10.1093/humrep/deaf184
Supplementary Data

Supplementary Table S2. Presence of typical symptoms.

	No E/A, n = 742	E/A, n = 293	P-value
**Dysmenorrhea**	510/742 (68.7)	268/293 (91.5)	<0.001*
**Dyspareunia**	121/741 (16.3)	127/293 (43.3)	<0.001*
**Dyschezia**	54/739 (7.3)	75/293 (25.6)	<0.001*
**Chronic pelvic pain**	216/740 (29.2)	147/291 (50.5)	<0.001*
**Dysuria**	15/741 (2.0)	39/292 (13.4)	<0.001*
**Hematochezia**	7/740 (0.9)	15/291 (5.2)	<0.001*
**Hematuria**	7/740 (0.9)	8/290 (2.8)	0.029*

E/A, endometriosis and/or adenomyosis. Numbers are given as n (%). Comparisons were made with the chi-square test. Due to missing values, percentages are counted as % of nb of women who answered the question. *$P > 0.05$ is considered indicating statistically significant difference.
