# Supplementary material for: Cumulative live birth rates under three consecutive IVF/ICSI treatment cycles are reduced in women with endometriosis and/or adenomyosis diagnosed by ultrasonography
Source: Hum Reprod. 2025 Sep 20;40(12):2332–41. doi: 10.1093/humrep/deaf184 (PMC12675419; doi:10.1093/humrep/deaf184)
Supplement: deaf184_Supplementary_Table_S3 [file deaf184_supplementary_table_s3.pdf]

OXFORD
human reproduction

Human Reproduction, 2025, 1–1
https://doi.org/10.1093/humrep/deaf184
Supplementary Data

Supplementary Table S3. Characteristics of adenomyosis in 102 women with direct features of adenomyosis.

	Number of women, n (%)
Type of feature[a]	
Lines and buds	76 (74.5)
Myometrial cysts	36 (35.3)
Hyperechogenic islands	52 (51.0)
Location of features[b]	
Inner	84 (82.4)
Middle	11 (10.8)
Inner to middle	44 (43.1)
Outer	11 (10.8)
Type of adenomyosis	
Focal	24 (23.5)
Diffuse	54 (52.9)
Mixed-type	24 (23.5)
Nb of direct features	
One	62 (60.8)
Two	26 (25.5)
Three	14 (13.7)
Severe (extent >50% of myometrium)	10 (9.8)
≥2 features	40 (39.2)

[a] Some women had more than one type of features.
[b] Some women had adenomyosis in multiple myometrial layers. Numbers are given as n (%).
