# Supplementary material for: Cumulative live birth rates under three consecutive IVF/ICSI treatment cycles are reduced in women with endometriosis and/or adenomyosis diagnosed by ultrasonography
Source: Hum Reprod. 2025 Sep 20;40(12):2332–41. doi: 10.1093/humrep/deaf184 (PMC12675419; doi:10.1093/humrep/deaf184)
Supplement: deaf184_Supplementary_Table_S4 [file deaf184_supplementary_table_s4.pdf]

OXFORD
human reproduction

Human Reproduction, 2025, 1–1
https://doi.org/10.1093/humrep/deaf184
Supplementary Data

Supplementary Table S4. Differences between women with endometriosis and/or adenomyosis who did or did not achieve live birth after maximum three consecutive IVF/ICSI treatments.

Characteristics	Live birth (+) (n = 156)	Live Birth (–) (n = 137)	P-value
Age (years)	32.1 (±3.8)	33.1 (±4.1)	0.074
BMI (kg/m^2)	23.3 (±3.7)	24.0 (±4.1)	0.382
Associated male infertility	38 (24.4)	35 (25.5)	0.814
Presence of leiomyomas	24 (15.4)	21 (15.3)	0.989
Associated endometriosis	130 (83.3)	102 (74.5)	0.062
Endometriosis phenotype			
Endometrioma	63 (40.4)	51 (37.2)	0.580
DE	105 (67.3)	78 (56.9)	0.067
DE with associated endometrioma	38 (24.4)	27 (19.7)	0.339
Patient's ovarian reserve:			
AMH (pmol/L)	21.1 (±20.1)	15.8 (±14.9)	<0.001*
AFC	17.6 (±8.1)	12.5 (±7.5)	<0.001*
Presence of direct features	46 (29.5)	56 (40.9)	0.041*
Concurrent endometriosis + adenomyosis	20 (12.8)	21 (15.3)	0.537
Location of adenomyosis[a]			
Outer myometrium	6 (3.8)	5 (3.6)	0.061
Middle	5 (3.2)	6 (4.4)	0.585
Inner-middle	20 (12.8)	24 (17.5)	0.085
Inner	50 (32.1)	34 (24.8)	0.102
Type of adenomyosis			
Focal	15 (9.6)	9 (6.6)	0.138
Diffuse	26 (16.7)	27 (19.7)	0.833
Mixed-type	9 (5.8)	15 (10.9)	0.214
Myometrial cysts	17 (10.9)	19 (13.9)	0.440
Lines and buds	34 (21.8)	42 (30.7)	0.084
Hyperechogenic islands	22 (14.1)	30 (21.9)	0.081
1 feature			
≥2 different features (direct + indirect)	60 (38.5)	66 (48.2)	0.073
≥2 direct features	14 (9.0)	26 (19.0)	0.039*
Indirect features present	79 (50.6)	88 (64.2)	0.019*
Indirect features without direct features	33 (21.2)	32 (23.4)	0.651
Extent of adenomyosis >50%	2 (1.3)	8 (5.8)	0.099
GnRH agonist protocol			
1st cycle	33/156 (21.2)	45/137 (32.8)	0.024*
2nd cycle	26/66 (39.4)	44/88 (50.0)	0.191
3rd cycle	16/22 (72.7)	45/62 (72.6)	0.989
ART procedure with ICSI			
1st cycle	47/155 (30.3)	38/125 (30.4)	0.989
2nd cycle	34/66 (51.5)	50/85 (58.8)	0.370
3rd cycle	16/22 (72.7)	40/58 (69.0)	0.743
Total FSH-dose (IU)			
1st cycle	1800 (900–6600)	2563 (1000–7200)	<0.001*
2nd cycle	2230 (827–7125)	3000 (200–7200)	0.005*
3rd cycle	3100 (920–7125)	3075 (500–7000)	0.768
Nb mature oocytes			
1st cycle	8 ±(8.2)	7 (±3.8)	<0.001*
2nd cycle	15.5 (±29)	6.2 (±4)	<0.001*
3rd cycle	8 (±9)	8 (±5.9)	0.347
Nb of GQE			
1st cycle	1 (0–3)	1 (0–3)	<0.001*
2nd cycle	1 (0–3)	1 (0–3)	0.241
3rd cycle	2 (1–6)	1 (0–7)	0.001*

(continued)

OXFORD

human reproduction

Human Reproduction, 2025, 1–1
https://doi.org/10.1093/humrep/deaf184
Supplementary Data

Supplementary Table S4. (continued)

Characteristics	Live birth (+) (n = 156)	Live Birth (–) (n = 137)	P-value
Blastocyst transfer			
1st cycle	57/129 (44.2)	29/84 (34.5)	0.160
2nd cycle	26/56 (46.4)	10/67 (14.9)	<0.001*
3rd cycle	3/20 (15.0)	4/42 (9.5)	0.524
FET			
1st cycle	43/50 (86.0)	29/38 (76.3)	0.243
2nd cycle	17/61 (27.9)	18/70 (25.7)	0.781
3rd cycle	6/22 (27.3)	12/54 (22.2)	0.639

AMH, anti-Müllerian hormone; AFC, antral follicle count; DE, deep endometriosis; FET, frozen embryo transfer; GQE, good-quality embryo; IU, international unit; nb, number.

[a] Some women may have adenomyosis in multiple locations. The data are presented as means (±SD), median (range), or n (%). Comparisons were made with the chi-square test, Fisher's exact test, Mann–Whitney *U*-test, or Student's *t*-test.

* $P > 0.05$ is considered indicating a statistically significant difference.

© The Author(s) 2025. Published by Oxford University Press on behalf of European Society of Human Reproduction and Embryology.
This is an Open Access article distributed under the terms of the Creative Commons Attribution License (https://creativecommons.org/licenses/by/4.0/), which permits unrestricted reuse, distribution, and reproduction in any medium, provided the original work is properly cited.
Human Reproduction, 2025, 00, 1–15
https://doi.org/10.1093/humrep/deaf184
Original Article
